# Cryopreservation alters contractile function of human induced pluripotent stem cell-derived cardiomyocytes

**DOI:** 10.1038/s41598-026-59927-4

**Published:** 2026-07-02

**Authors:** Kathrin Kowalski, Benita Haß, Judith Montag, Joachim D. Meißner, Jana Teske, Robert Zweigerdt, Theresia Kraft

**Affiliations:** 1 Institute of Molecular and Cell Physiology, Hannover Medical School (MHH), Hannover, Germany; 2 Department of Human Medicine, Medical School Berlin (MSB), Berlin, Germany; 3 Department of Cardiothoracic, Transplantation and Vascular Surgery (HTTG), Leibniz Research Laboratories for Biotechnology and Artificial Organs (LEBAO), Hannover Medical School (MHH), Hannover, Germany

**Keywords:** Cryopreservation, hiPSC-CMs, Contraction parameters, Long-term culture, Biological techniques, Biotechnology, Cell biology, Developmental biology, Stem cells

## Abstract

Advanced protocols are available for efficient generation of large quantities of human induced pluripotent stem cell-derived cardiomyocytes (hiPSC-CMs). Nevertheless, hiPSC-CMs show large batch to batch variations as well as fetal-like phenotype. Cryopreservation enables long-term storage of batches, leading to increased consistency and reproducibility of data while improving maturation. However, controversial data regarding comparability of fresh and cryopreserved hiPSC-CMs have been reported. Here, we compared fresh and cryopreserved hiPSC-CMs, demonstrating that both cryopreservation media (CryoStor^®^CS10 and KnockOut Serum Replacement) had a comparable recovery rate (CS10: 39%, KSR: 46%) and similar proportion of CMs in long-term culture. Cryopreservation altered cell morphology (increased cell area and shorter or longer sarcomere length) and contractile parameters (faster time to peak and half relaxation time and higher or shorter contraction amplitude) of recovered hiPSC-CMs with only slight changes in sarcomeric gene and protein expression. Some differential effects of both cryo-media on CM structure and function were observed. The data indicate an influence of cryopreservation on cell morphology as well as on contraction parameters that should be considered in downstream applications.

## Introduction

Human induced pluripotent stem cell-derived cardiomyocytes (hiPSC-CMs) are an established in vitro model to study human heart development, cardiovascular diseases, neuro-cardiac interactions, or to perform drug screening^1–4^. For example, hiPSC-CMs are used as a model to study effects of specific mutations causing hypertrophic cardiomyopathy^3,5–10^. Moreover, recent pilot studies successfully used tissue-patches derived from hiPSC-CMs for myocardial repair in patients with myocardial infarction^11–13^. Therefore, well-characterized and standardized batches of hiPSC-CMs are required. Efficient generation of large quantities of relatively pure hiPSC-CM cultures is possible due to advanced protocols^14–17^. Nevertheless, persistent limitations are related to variability between differentiations as well as limited maturation with regard to transcriptome and proteome, especially sarcomeric gene and protein expression, and to morphology and function^3,9,18–23^ that indicate non-mature cardiomyocyte characteristics of hiPSC-CMs. Different approaches are reported to enhance maturation of hiPSC-CMs, e.g. long-term cultivation, culture medium composition, extracellular matrices, micro-grooved scaffolds or co-cultures with other cell types^1,13,24–26^. Furthermore, cryopreservation was shown to promote maturation of hiPSC-CMs related to ventricular markers towards a more adult-like phenotype^27–29^ and to improve consistency in functional assays with similar results^30^. On the other hand, cryopreserved hESC-CMs have been reported to exhibit damaged cell and nuclear membranes, faster beating frequency and loss of mitochondrial integrity^31^. Although this sounds contradictory, physical injury could lead to DNA damage response or oxidative stress and this could affect downstream pathways involved in maturation processes like mTOR and p53^32,33^. Nevertheless, cryopreservation of up-scaled hiPSC-CMs batches could be beneficial for several approaches as well as cooperation between laboratories.

However, previous studies only assessed cryopreserved hiPSC-CMs^30,31,34^ or compared fresh with cryopreserved hiPSC-CMs only immediately before and within a few days after thawing^27,35,36^, demonstrating inconsistent effects of cryopreservation on contractility compared to fresh cultures^27,28,36^. Thus, it remains unknown how cryopreserved hiPSC-CMs adapt upon longer cultivation with regard to contractile parameters. It is well known that longer cultivation of hiPSC-CMs supports maturation of stem cell-derived CMs^3,7,21,37,38^. Therefore, we compared cultures recovered after cryopreservation with two standard, commercially available cryopreservation media, CryoStor^®^CS10 (CS10) and KnockOut Serum Replacement (KSR)^29^, with corresponding freshly cultured hiPSC-CMs not only at day 10 but also at day 35 of culture on laminin-coated glass coverslips. Both cryopreservation media include the cryoprotective agent dimethyl sulfoxide (10%), are serum-free, both should reduce cell death and improve cell viability and function after thawing. We addressed the questions whether and how cryopreservation media CS10 and KSR (a) affect viability of hiPSC-CMs and the ratio of cardiomyocytes to non-cardiomyocytes in recovered cultures, (b) influence transcriptional activity of sarcomeric genes *MYH6* (coding for α-myosin heavy chain, α-MyHC), *MYH7* (coding for β-MyHC), *MYBPC3* (coding for cardiac myosin binding protein C, cMyBP-C) and *TNNI3* (coding for cardiac troponin I, cTnI) in recovered hiPSC-CMs, (c) influence expression of the respective sarcomeric proteins α- and β-MyHC, cMyBP-C and cTnI in recovered hiPSC-CMs and (d) influence CM-morphology and contractile properties of recovered hiPSC-CMs, all on single cell level.

Recovered hiPSC-CMs showed no major changes of transcriptional activity of sarcomeric genes and their respective protein levels. However, we detected changes in cell morphology as indicated by analysis of cell area and sarcomere length. Furthermore, we found altered contractile properties of cryopreserved hiPSC-CMs compared with freshly cultured cells after 35 days on laminin-coated glass cover slips and some diverging effects of both cryopreservation media on CM survival, morphology, gene expression and function.

## Results

### Both cryopreservation media have a comparable recovery rate and similar proportion of CMs in long-term culture

**Fig. 1:**
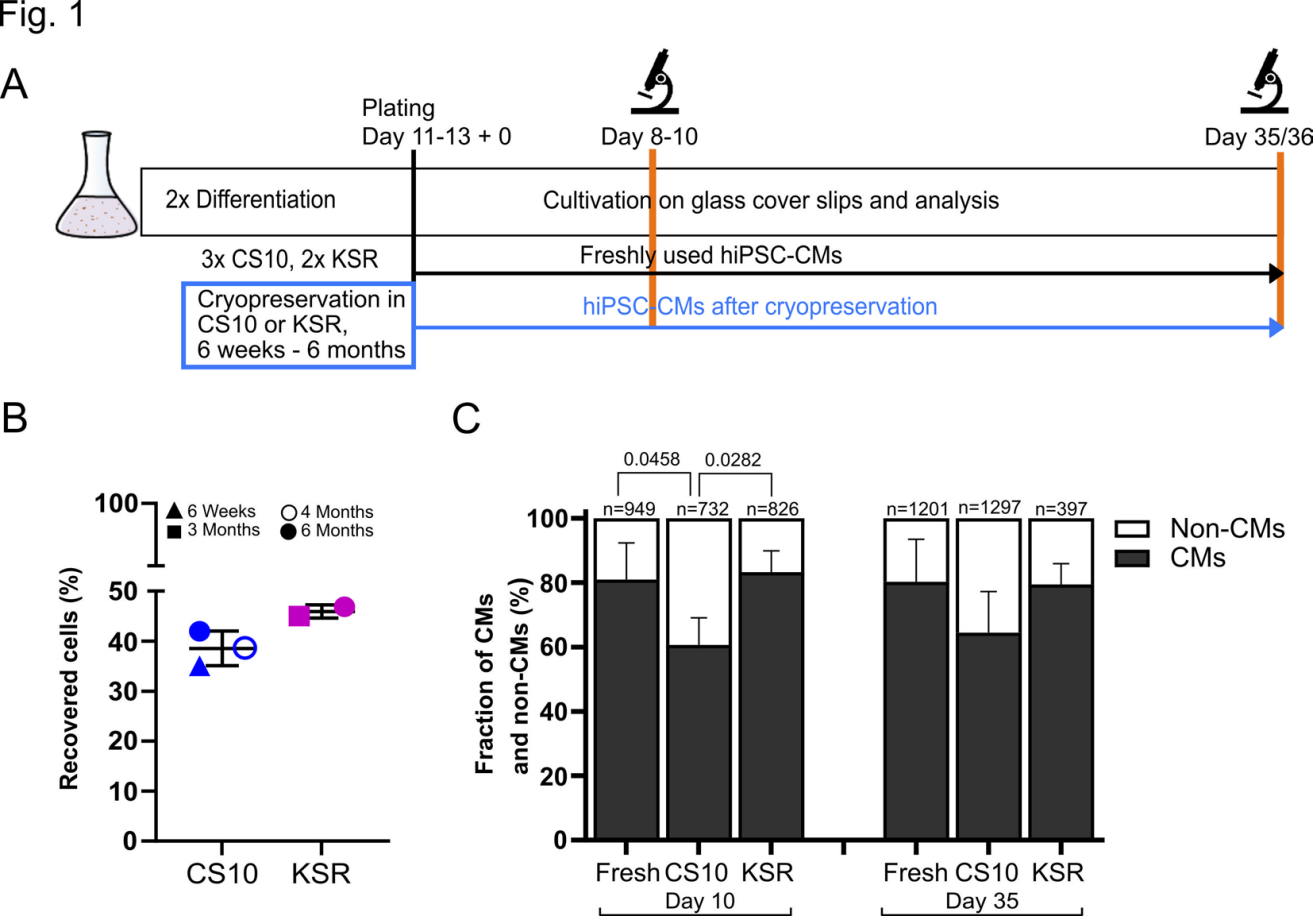
Effect of cryopreservation on recovery rate and cardiomyocyte content. (**A**) Schematic outline of the cultivation protocol. hiPSC-CMs were differentiated in suspension culture until day 11–13. Afterwards hiPSC-CMs were seeded on laminin-coated glass cover slips (+ 0) immediately or after cryopreservation in CS10 (3 vials) or KSR (2 vials) medium for six weeks up to six months before plating. Cells were analyzed at day 8–10 and 35/36. (**B**) Recovery rate (percentage of recovered living cells, total number of cryopreserved cells set to 100%) of cells after cryopreservation in CS10 (3 vials) and KSR (2 vials) media for indicated times. Unpaired t test. (**C**) Percentage of cardiomyocytes (CMs) and non-CMs in fresh and recovered cryopreserved cultures. Expression of myosin was analyzed by immunofluorescence using antibodies against α- and β-MyHC. Fraction of CMs was calculated as percentage of α- and β-MyHC positive cells, with of total cell number determined by bright field microscopy and nuclei counting set to 100%. Mean ± SD (**B**,**C**). One-way ANOVA with Tukey’s post-hoc test. n= number of analyzed cardiomyocytes from two differentiations.

To test the effect of cryopreservation on differentiated hiPSC-CMs, we compared freshly cultured hiPSC-CMs with hiPSC-CMs that were recovered after cryopreservation for six weeks up to six months from same differentiations. For cryopreservation of hiPSC-CMs two standard cryopreservation media, CryoStor^®^CS10 (CS10) and KnockOut Serum Replacement (KSR), were used. Freshly differentiated and recovered hiPSC-CMs, respectively, were cultivated on laminin-coated glass cover slips and analyzed on day 10 and day 35 (Fig. 1A). A major concern regarding cryopreservation is cell viability, as ice crystallization during the freezing process may damage the cell membrane and lead to decreased cell recovery^31,39,40^. To assess cell viability, we determined the recovery rate directly after thawing by using trypan blue exclusion. After cryopreservation for 3 and 6 months in KSR medium, on average 46% viable cells could be detected (Fig. 1B). After 6 weeks up to 6 months cryopreservation in CS10 medium, recovery rate was not significant lower with on average 39%. In summary, only less than half of the frozen cells could be cultivated again after cryopreservation in short and long term stored hiPSC-CM cultures.

So far, it is essentially not possible to generate hiPSC-CM cultures containing exclusively cardiomyocytes^16,35,41^, although we analyzed cultures with 92% and 94% CM purity. To reveal whether cryopreservation affects the fraction of non-cardiomyocytes (non-CMs), we analyzed the expression of CM-specific myosin heavy chain isoforms (MyHC) by immunofluorescence staining in single hiPSC-CMs. In fresh cultures, the percentage of CMs did not change over time (81% at day 10 and 80% at day 35; Fig. 1C). In CS10-cryopreserved cultures, ten days after thawing CM fraction of CS10 cultures was 61% and remained low with 64% at day 35, demonstrating a significant reduction only at day 10 compared to freshly analyzed and also to KSR-cryopreserved cultures. In KSR-cryopreserved cultures, CM fraction was similar to the freshly used cultures (83% at day 10 and of 79% at day 35) (Fig. 1C).

### Cryopreservation alters cell morphology and sarcomere length

**Fig. 2:**
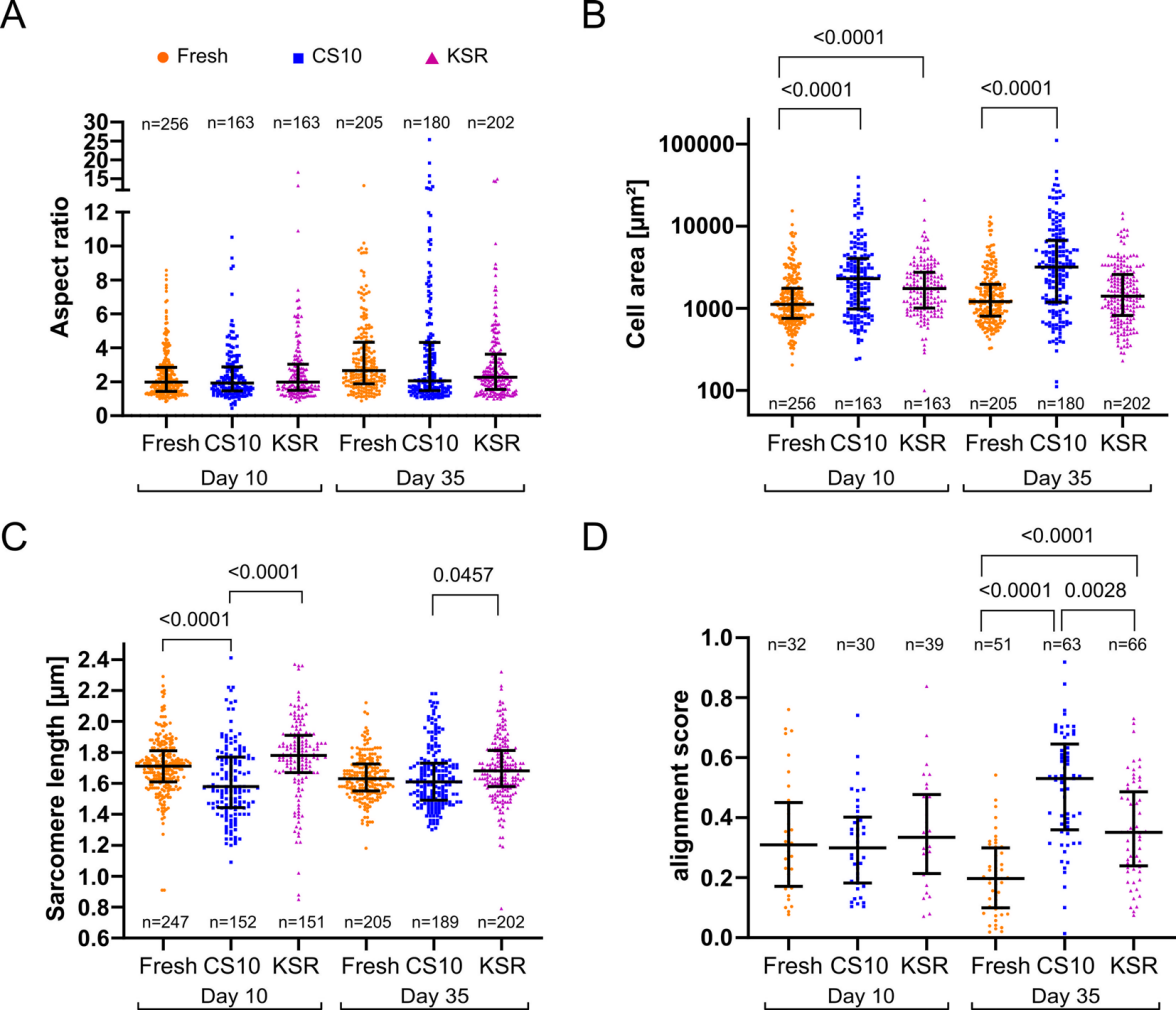
Effect of cryopreservation on cell morphology and sarcomere length. (**A**) Aspect ratio (maximal length/maximal width), (**B**) cell area, (**C**) sarcomere length and (**D**) alignment score of fresh and cryopreserved cardiomyocytes were analyzed by using Image J. Median ± interquartile range, Kruskal-Wallis test with Dunn’s post-hoc test, significant differences with *p* < 0.05. n= number of analyzed hiPSC-CMs from two differentiation, except for alignment score (*N* = 1).

It was previously shown that cryopreservation led to increased cell size of hiPSC-CMs^28^. Here, the morphology of recovered hiPSC-CMs from both cryopreservation media, as indicated by aspect ratio (maximal length/maximal width), was similar to fresh cells at day 10 (fresh: 1.99; CS10: 1.92; KSR: 1.898, Fig. 2A) and day 35 (fresh: 2.66; CS10: 2.06; KSR: 2.27).

Furthermore, we observed a significant increase in cell area of hiPSC-CMs cryopreserved in both media compared with fresh cells at day 10 (fresh: 1119 µm^2^; CS10: 2288 µm^2^; KSR: 1742 µm^2^; Fig. 2B). Upon longer cultivation (day 35), this difference was abolished in KSR-cryopreserved hiPSC-CMs, however, CS10-cryopreserved hiPSC-CMs maintained a significant increase in cell area (fresh: 1202 µm^2^; CS10: 3167 µm^2^; KSR: 1401 µm^2^).

Moreover, sarcomere length of fixed hiPSC-CMs was altered upon cryopreservation, with both media showing different effects. In freshly cultivated hiPSC-CMs, sarcomere length was slightly reduced over time, from 1.71 μm at day 10 to 1.63 μm at day 35 (Fig. 2C). CS10-cryopreserved hiPSC-CMs showed a significantly shorter sarcomere length of 1.58 μm at day 10, which increased to 1.61 μm at day 35 and was thus comparable to freshly cultivated hiPSC-CMs at later time point. KSR-cryopreserved hiPSC-CMs showed similar sarcomere length compared to fresh cultures at both time points (1.78 μm at day 10 and 1.68 μm at day 35), but exhibit significantly larger sarcomere length compared to CS10 cultures at day 10 and day 35. We also investigated myofibrillar alignment based on immunofluorescence images as another measure of maturity^42^. At day 10, average orientation of myofibrils in cryopreserved hiPSC-CMs was similar to fresh cultures (fresh: 0.3096; CS10: 0.2992; KSR: 0.3343), but at day 35 both cryopreserved hiPSC-CM cultures showed a significantly increased alignment score with the highest for CS10-culture that was also significantly higher than for KSR-CMs, indicating more aligned myofibrils (fresh: 0.1969; CS10: 0.5308; KSR: 0.3511) (Fig. 2D).

### Only slight changes in sarcomeric gene and protein expression in recovered hiPSC-CMs

**Fig. 3:**
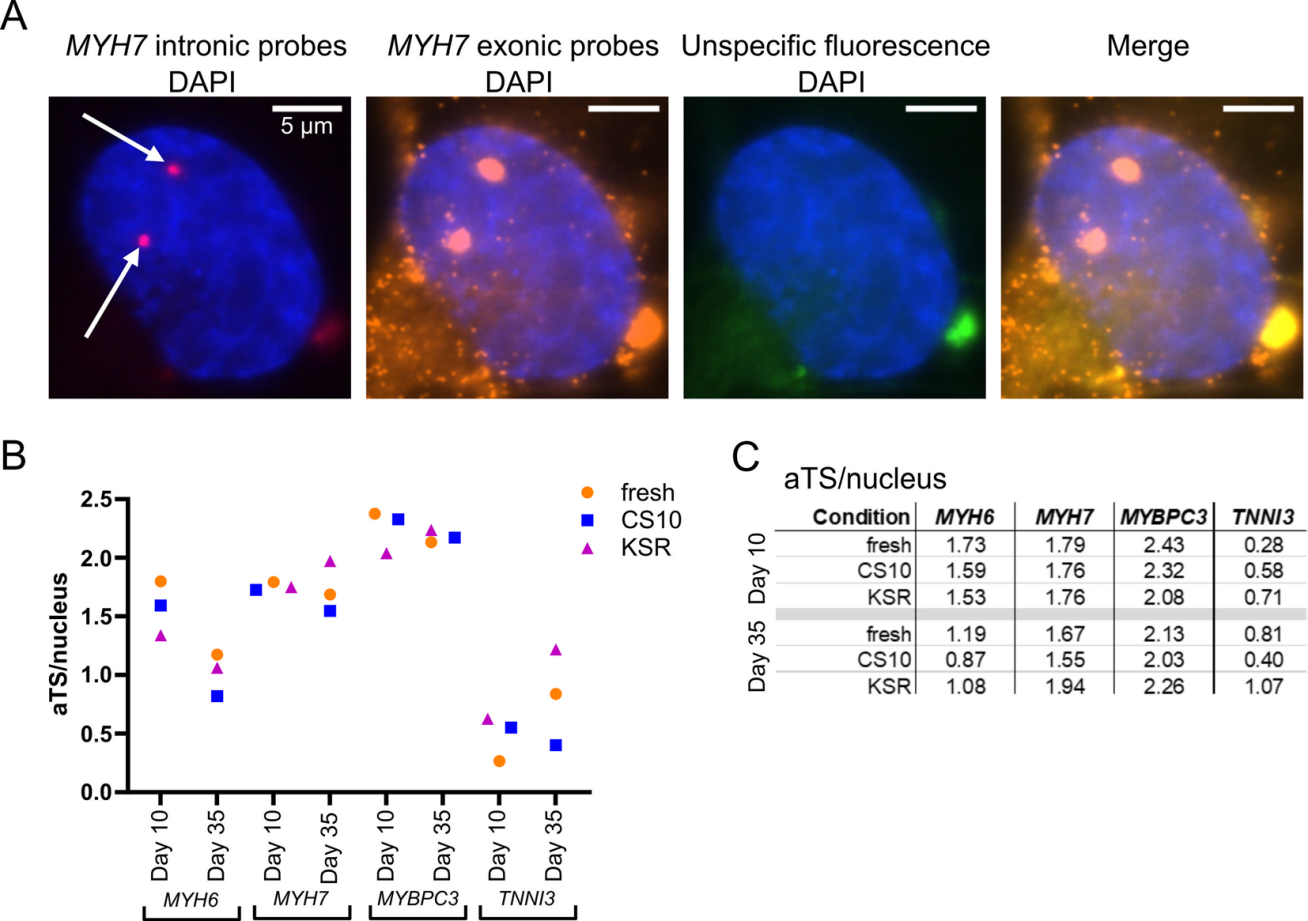
Effect of cryopreservation on sarcomeric gene transcription. (**A**) Single molecule RNA fluorescence in situ hybridization analysis of transcriptional activity using specific *MYH7* intronic (red) and exonic (orange) probe sets. GFP-channel was used for unspecific fluorescence. Representative hiPSC-CM nucleus (blue, DAPI) with two active transcription sites (arrows) of *MYH7* at day 10 on laminin-coated glass cover slip recovered from cryopreservation in CS10 media for 4 months. Cytoplasmic *MYH7*-mRNA expression defines cell as cardiomyocyte. (**B**) Transcriptional activity (number of aTS/nucleus) of indicated genes at days 10 and 35 of fresh, CS10- and KSR-hiPSC-CMs, respectively. Mean from 3 (CS10) and 2 (KSR) vials, respectively, from 2 differentiations. (**C**) Number of aTS/nucleus.

Previously, it was demonstrated that freezing and thawing of hiPSC-CMs supports their maturation, and this was indicated by expression of adult isoforms of sarcomeric genes and proteins^27,28^. To address this point, we used single molecule RNA fluorescence in situ hybridization (smRNA-FISH) to analyze the transcriptional activity of four sarcomeric genes (*MYH6*, *MYH7*, *MYBPC3*, *TNNI3*) in fresh and cryopreserved hiPSC-CMs. No significant differences in the number of active transcription sites (aTS) per nucleus between fresh and cryopreserved CMs were detected at both time points, except *MYH6* and *TNNI3* (Fig. 3A-D). At day 10, KSR-cryopreserved hiPSC-CMs showed a significantly lower transcriptional activity for *MYH6* compared to fresh hiPSC-CMs (*p* = 0.0460). Interestingly, *TNNI3*, which had rather low transcriptional activity in fresh cultivated CMs at day 10, showed more than two fold but not significantly higher number of aTS per nucleus for cryopreserved hiPSC-CMs (fresh: 0.28; CS10: 0.58; KSR: 0.71 aTS per nucleus, respectively, *p* > 0.05 for all). At day 35, for fresh and KSR-cryopreserved hiPSC-CMs transcriptional activity of *TNNI3* was further substantially but not significantly increased, while it was lower in CS10 hiPSC-CMs. For KSR-cryopreserved vs. CS10-cryopreserved CMs the difference at day 35 was significant (*p* = 0.0011).

Transcriptional activity of *MYBPC3* was the highest in all conditions, as observed in human left ventricular tissue before^43,44^.

**Fig. 4:**
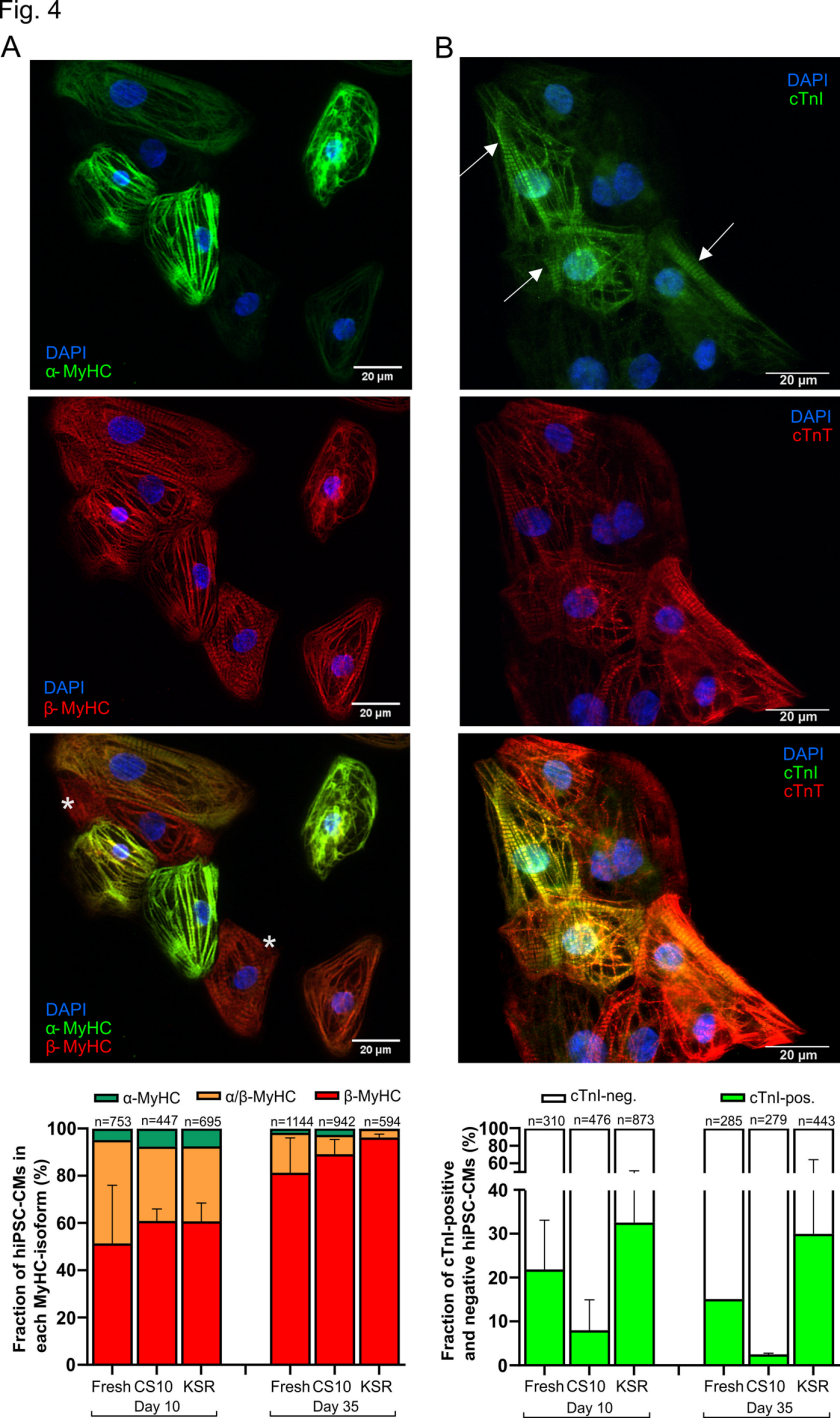
Effect of cryopreservation on sarcomeric protein expression analyzed by single cell IF. (**A**) Representative immunofluorescence (IF) images of CS10-cryopreserved hiPSC-CMs (day 10) stained with specific antibodies against α- and β-MyHC, representing examples of α-MyHC (green), β-MyHC (red) expressing CMs and CMs with mixed α-/β-MyHC (orange) expression. hiPSC-CMs marked with * represent exclusively β-MyHC expressing CMs. Percentage of CMs in each MyHC-category at day 10 and 35 in fresh, CS10- and KSR- hiPSC-CMs, based on sarcomere staining only. (**B**) Representative IF images of KSR-cryopreserved hiPSC-CMs (day 10) stained with specific antibody against cTnI (white arrows: cTnI-positive CMs). Only cTnT-expressing (red) cells were analyzed. Percentage of cTnI-positive (based on sarcomere staining only) and negative fresh, CS10- and KSR-hiPSC-CMs at day 10 and 35. (**A**,**B**) DAPI for nuclear staining. Scale bar 20 μm. Mean ± SD, n=number of analyzed cardiomyocytes from 3 (CS10) and 2 (KSR) vials, respectively, from 2 differentiations. One way ANOVA test showed no significant differences.

To assess the influence of cryopreservation on sarcomeric proteins directly, expression of α- and β-MyHC as well as cMyBP-C and cTnI in fresh and cryopreserved hiPSC-CMs was analyzed at days 10 and 35 of culture by single cell immunofluorescence (IF). As reported previously, a shift towards a predominance of exclusively β-MyHC expressing hiPSC-CMs could be observed over time^37,45^. This was also found in cryopreserved hiPSC-CMs and no significant differences were detected between the approaches at day 10 and 35 (Fig. 4A). However, a minor shift towards a higher fraction of exclusively β-MyHC expressing hiPSC-CMs was observed for both cryopreservation media compared to fresh CMs at both time points.

The percentage of cTnI-positive CMs in fresh cultures remained fairly stable over time (22% and 15%, respectively) (Fig. 4B). CS10 cultures showed rather few cTnI-positive CMs at day 10 and day 35 (8% and 2%, respectively). KSR-cryopreserved CMs showed the highest fraction of cTnI expressing CMs (33% and 30%, respectively). Neither these differences nor the differences between the two cryopreservation media were significant at both time points. Cardiac MyBP-C level were also neither different between conditions nor changed much over time (data not shown).

### Contractile parameters are altered after cryopreservation

**Fig. 5:**
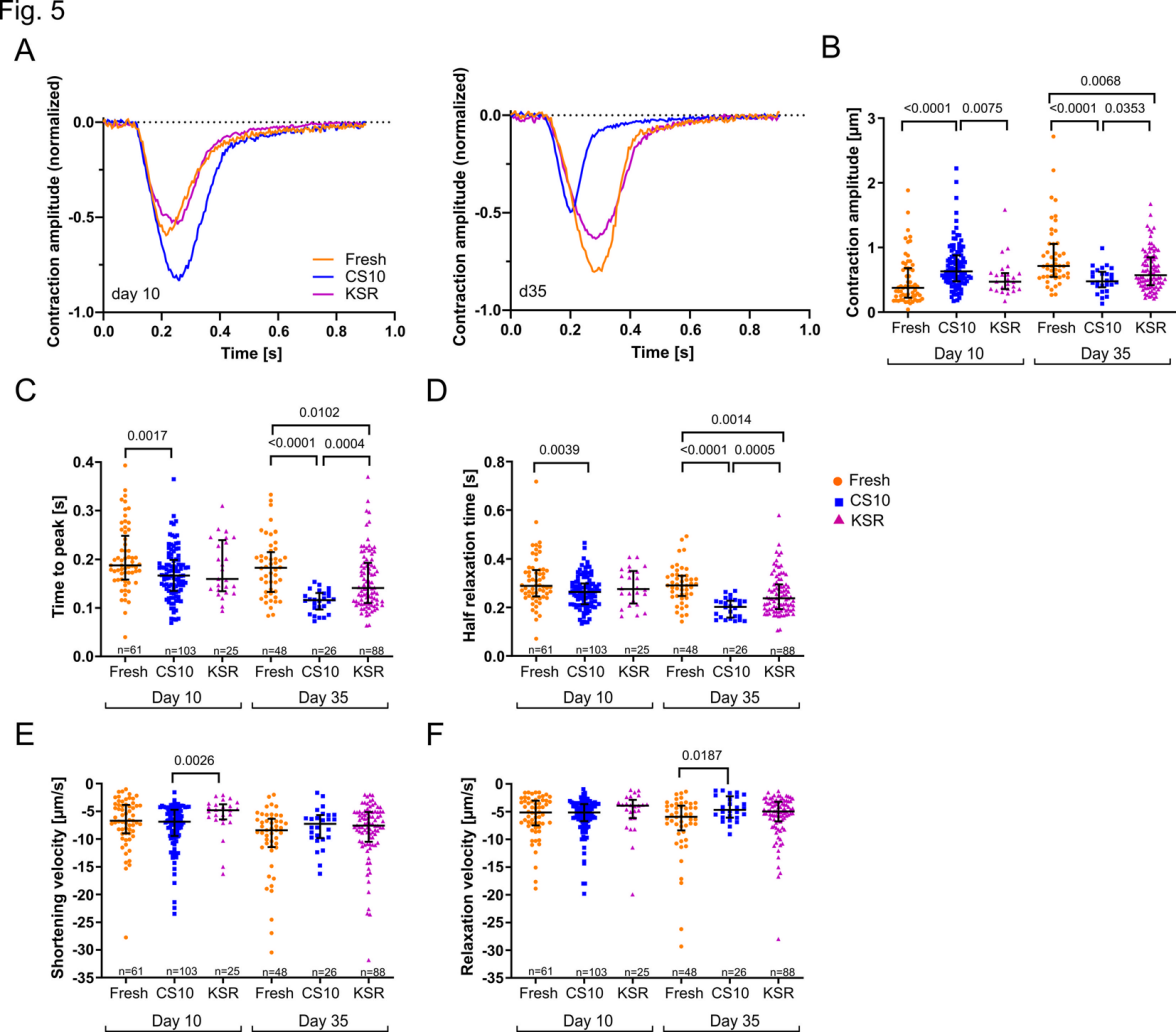
Effect of cryopreservation on contractile parameters after cryopreservation. (**A**) Representative twitches of fresh (orange), CS10- (blue) and KSR- (purple) hiPSC-CMs. CMs were paced at 1 Hz at 37 °C. (**B**) Contraction amplitude, (**C**) time to peak, (**D**) half relaxation time (hrt) of twitches, (**E**) shortening velocity and (**F**) relaxation velocity. Median ± interquartile range, Mann-Whitney test, significant differences with *p* < 0.05, n=number of analyzed cardiomyocytes from 3 (CS10) and 2 (KSR) vials, respectively, from 2 differentiations.

Previous studies showed inconsistent effects of cryopreservation on contraction characteristics of hiPSC-CMs^27,28,36^. To test if cryopreservation affects CM function, contraction parameters of single hiPSC-CMs were analyzed with a MyoCam (IonOptix) setup. Twitches were recorded from single CMs electrically stimulated at 1 Hz for 1 min using the edge detection method. At day 10, twitches from fresh and KSR-cryopreserved hiPSC-CMs were comparable with regard to twitch amplitude, time to peak (ttp) and half-relaxation time (hrt) (Fig. 5A–D). Interestingly, CS10-cryopreserved hiPSC-CMs showed significantly increased twitch amplitude and significantly shorter ttp and hrt. At day 35, both CS10- and KSR-cryopreserved hiPSC-CMs showed a significantly reduced twitch amplitude, shorter ttp and hrt compared with fresh CMs. Twitch amplitude, ttp and hrt were significantly different between CS10- and KSR-cryopreserved hiPSC-CMs at day 35. Shortening and relaxation velocities were comparable in all conditions at both time points (Fig. 5E, F). Only significant differences were found between shortening velocity of CS10- vs. KSR-cryopreserved CMs at day 10 and relaxation velocity of fresh vs. CS10-cryopreserved CMs at day 35.

## Discussion

In this study we aimed to elucidate the impact of cryopreservation of hiPSC-CMs on morphology, sarcomeric gene and protein expression, as well as contraction parameters. For this purpose, we compared hiPSC-CMs cryopreserved in two different media (CS10 and KSR) with their freshly cultivated counterparts from the same differentiations after cultivation on laminin-coated glass cover slips focusing on single-cell analysis at day 10 and 35 of culture.

The recovery rate of living cells of 39% for CS10- and 46% for KSR-cryopreserved cells was similar to or lower than those from other reports on cryopreserved hiPSC-CMs or human embryonic stem cell-derived (hESC-)CMs^27,31,35,36,46^. With regard to recovery rate cryopreservation with CS10 is less favorable in our hands, although other groups observed a recovery rate of 70–85%^35,46,47^. A better recovery rate of KSR-cryopreserved cells compared to CS10 was also shown previously^29^. Although both cryopreservation media should reduce cell death and improve cell viability and function after thawing, the recovery rate is relatively low but the fraction of CMs in cryopreserved cultures was constant and similar to fresh cultures. Despite low vial number, duration of cryopreservation seems to have no influence on recovery rate as shown before^27,36,46^.

We also assessed whether cryopreservation influences the fraction of cardiomyocytes in the culture. Starting from a high cardiomyocyte purity (94% and 92%) after differentiation verified by FACS analysis, we observed a ~ 20% reduction in fraction of cardiomyocytes in random fields of view after immunofluorescence staining, which remained stable over time. This observation is in line with previous studies demonstrating a general 10–20% decrease in proportion of CMs after plating^28,36^. Despite improved hiPSC-CM differentiation protocols, some other cell types like cardiac fibroblast or cardiac progenitor cells could remain^48^, which could lead to non-CM expansion. The reduced fraction of CMs could also be due to CM death or dedifferentiation. This could be examined e.g. by FACS or apoptosis markers. Additionally, cell cycle components are changing during hiPSC-CM culture time as well as myofibril formation conflicts with cell cycle maintenance^49^. Nevertheless, cryopreservation in KSR-medium did not affect fraction of CMs in the culture. In contrast, CS10-cryopreservation led to a decrease in CMs upon cultivation. Since the fraction remained stable over time, it seems likely that a higher proportion of CMs does not survive freezing in CS10-medium compared to other cell types, rather than induction of a higher proliferation of non-CMs in these cultures. Thus, for studies which require a high and reproducible fraction of CMs, KSR seems favorable in our study. For future studies it could be useful to elucidate which cell types preferably survive CS10-cryopreservation, however, this is beyond the scope of this study.

Cryopreservation of hiPSC-CMs in our hands leads to loss of approximately half of the cells. Whether this loss also negatively affects the overall fraction of cardiomyocytes depends on the freezing medium that seems to affect hiPSC-CMs differently. Cardiomyocyte survival seems to be more affected in CS10 medium.

Cells recovered after cryopreservation were shown to exhibit an upregulation of cell cycle and division genes^15,36,47^. Analysis of cell morphology revealed increased cell size and changed sarcomere length for both media. Increased cells size of cryopreserved hiPSC-CMs was also previously observed^28^ as well as for CM aggregates after CS10-cryopreservation^47^. In addition, cryopreserved CMs showed a higher alignment score upon longer cultivation, which was most pronounced in CS10-cryopreserved cells. Nevertheless, it’s still to be clarified whether hypertrophic markers are upregulated.

Analysis of transcriptional activity using mRNA-fluorescence in situ hybridization showed time-dependent alterations, e.g., a decrease in *MYH6* transcription in both, fresh and cryopreserved hiPSC-CMs on day 35. Studies addressing mRNA-expression by qPCR have shown that cryopreservation in KSR or BamBanker cryopreservation medium can lead to increased expression of *MYH7* and *TNNI3*^27,28^. In our study, we did not detect significant alterations, however, we could detect a trend to increased transcription of both genes and increased fraction of exclusively β-MyHC expressing or cTnI positive CMs in KSR-cryopreserved hiPSC-CMs. In case of β-MyHC the number of exclusively β-MyHC expressing CMs was already high and increased further with cryopreservation. For cTnI we observed higher inter-batch variances, presumably due to the overall lower cTnI-expression. The data do not indicate an effect of cryopreservation on maturation at level of sarcomeric gene expression. Faster relaxation parameter have been shown in a model of higher cTnI expression in KSR-cryopreserved hiPSC-CMs^50^. Interestingly, in line with no changes in relaxation velocity, KSR-cultures showed no significant change in the percentage of cTnI-positive hiPSC-CMs. Taken together, our data indicate that cryopreservation only induces minor alterations in sarcomeric gene and protein expression at least on single cell level. We cannot rule out effects of possible differential phosphorylation of sarcomeric proteins in cryopreserved CMs.

Controversial reports exist on the effect of cryopreservation on hiPSC-CM function. On the one hand cryopreservation of hiPSC-CMs was reported to have no effect on contractile function using KSR medium^27^, on the other hand alterations in functional characteristics like contraction velocity and relaxation velocity were observed with BamBanker medium^28^. In our study, CS10 and KSR cryopreservation media reduced the contraction amplitude, time to peak, and half relaxation time at day 35. Reduced ttp and hrt most likely result from the reduced contraction amplitude of cryopreserved hiPSC-CMs. A similar contraction amplitude of fresh and cryopreserved hiPSC-CMs was observed by van den Brink et al.^27^. Yet, in our experiments no consistent relationship between cell size, myofibrillar alignment and contraction amplitude was found, indicating that changes in cell size do not underlie decreased contraction amplitude.

Alpha and β-MyHC mainly determine contraction force and shortening velocity of sarcomeres^51^, and myosin isoform composition as well as the TnI-isoform influence cross bridge kinetics^52,53^. Here we observe mainly structural and functional alterations in cryopreserved cells, whereas sarcomeric gene and protein expression is mostly unaffected. In comparison to fresh cultures cryopreservation led to increased cell area and alignment score upon longer cultivation, most pronounced in CS10-cultures. This indicates that structural improvement in CS10-cryopreserved hiPSC-CMs was not directly translated to increased contraction. This hints further cellular alterations in these cells, which might also affect outcome of e.g. drug screening experiments. It should be noted that structural as well as functional alterations were less pronounced or not detectable in KSR-cryopreserved hiPSC-CMs, which thus show a higher comparability to fresh cultures.

The deeper analysis of such alterations was beyond the scope of the study and is associated with some limitations. Here, we only analyzed two differentiations of a single hiPSC-CM line, limiting generalizability. Different cell lines could show different outcomes for the same experimental setup as shown before^27,36^. To rule out heterogeneity in differentiations or cell lines causing reported differences here, our data could be verified by increasing the number of differentiations per cell line and with other cell lines. In addition, conclusions from the effects of cryopreservation on cell recovery might be limited by low vial numbers. Furthermore, analysis of Ca^2+^ transients, Ca^2+^ handling proteins, excitation-contraction coupling as well as sarcomeric protein’s phosphorylation status could help to explain observed differences in contractile parameters. Moreover, analysis of mitochondrial function and electrophysiology could provide a more comprehensive analysis of the effects of cryopreservation on CM maturation and function, respectively. Other analytical methods like transcriptomics or proteomics might provide clues to mechanistic insights in the observed effects of cryopreservation. Additionally, it remains to be investigated, whether effects of cryopreservation mask sarcomeric disease effects or if cryopreserved hiPSC-CMs react different in drug screening assays due to changed protein features. Here, we used a 2D hiPSC-CM model and it remains to be clarified how cryopreserved hiPSC-CMs behave as a monolayer, in a 3D model or under mechanical stress.

In summary, our study shows that hiPSC-CMs recovered after cryopreservation have similar sarcomeric gene and protein expression compared to freshly used cultures, with some differential effects of both cryopreservation media. KSR cryopreserved cultures showed a higher CM content compared with CS10 cryopreserved cultures, and less differences in contractile parameters compared with fresh CMs.

Although our conclusions have to be interpreted carefully, cell morphology as well as some contraction parameters are altered by cryopreservation. This should be considered when comparing data from studies performed with frozen and fresh hiPSC-CMs and especially in downstream applications.

## Methods

### Cell culture

hiPSC-CMs were differentiated from Phoenix cell line hHSC_Iso4_ADCF_SeViPS2 (MHHi001-A, female donor^54^ in two independent rounds of differentiation (differentiation at passage 43 and 44, respectively). Cells were routinely screened for Mycoplasma using the MycoStrip^®^ Myocoplasma Detection Kit (InvivoGen) or MycoAltert^®^ Mycoplasma Detection Kit (Lonza). No Mycoplasma contaminations have been detected. Pluripotency was assessed by flow cytometry using antibodies against NANOG (Miltenyi Biotec), OCT-3/4 (Santa Cruz Biotechnology), SSEA-4 (Development Studies Hybridoma Bank) and TRA-1-60 (Abcam)^54^. The hiPSC-CMs were generated by using an established protocol of WNT pathway modulation^16,41^. Briefly, hiPSC-CMs differentiation was performed in suspension culture as cardiac bodies (CBs) from frozen stocks of Phoenix cell line hHSC_Iso4_ADCF_SeV-iPS2 in Erlenmeyer flasks until day 11 to 13. Cardiomyocyte content was assessed at day of cryopreservation/plating of fresh CMs by flow cytometry using specific antibodies against the sarcomere markers cardiac troponin T (cTnT, Invitrogen), sarcomeric α-actinin (SA, Merck), β-myosin heavy chain (β-MyHC, Merck) and meromyosin portion of myosin heavy chain (MF20, Hybridoma Bank). Cardiomyocyte content was 92.1% and 93.7%, respectively. CBs were dissociated by using STEMdiff Cardiomyocyte Dissociation Kit (STEMcell Technologies). For freshly used cultures, cells were seeded at a density of 15,000 or 30.000 cells per 18 mm cover slip on laminin-coated (20 µg/ml; Merck) glass coverslips. For cryopreservation, 3.9 or 5.0 × 10^6^ cells/mL (differentiation 1 and 2, respectively) from the same batches as fresh hiPSC-CMs were frozen at − 150 °C in CryoStor^®^ CS10 (STEMcell Technologies) or KSR medium (Gibco Thermo Fisher Scientific, + 10% DMSO, Rho-associated protein kinase (ROCK)-inhibitor Y-27632 (10 µM, Tocris Bioscience) and Pluronic-F68 (10 µL/mL, Gibco, Thermo Fisher Scientific)), respectively. Cryopreserved cells were again counted after thawing followed by seeding on laminin-coated (0.02 mg/mL in PBS, Merck) glass cover slips (15,000 or 30,000 cells). All cells were cultivated for the first 24 h in IMDM Glutamax (Gibco, Thermo Fisher Scientific) supplemented with 1 mmol/L L-glutamine, 1% nonessential amino acids (both Gibco, Life Technologies), 0.1 mmol/L β-mercaptoethanol (Thermo Fisher Scientific) and 10 µM ROCK inhibitor Y-27,632, 1 U/mL penicillin-streptomycin (Gibco, Thermo Fisher Scientific) and 10% FBS (HyClone defined fetal bovine serum, Cytiva). After 24 h the cell culture medium was changed to bSF (basic serum free) medium consisting of Dulbecco’s modified Eagle’s medium (DMEM; Gibco, Thermo Fisher Scientific) supplemented with 1 mmol/L L-glutamine, 1% nonessential amino acids (both Gibco, Thermo Fisher Scientific), 1 U/mL penicillin-streptomycin (Gibco, Thermo Fisher Scientific), 17 µg/ml sodium selenite, 11 µg/ml transferrin and 50 µg/ml human insulin (all Sigma-Aldrich). Medium was exchanged twice a week including monitoring culture with a microscope. Cells were cultivated at 37 °C and 5% CO_2_. Analysis days were day 10 or 35 on cover slips, except for contraction analysis. For contraction analysis hiPSC-CMs were 8–10 days and 35–36 days cultivated on cover slips.

### Freezing and thawing of hiPSC-CMs

Differentiated CBs were spun down (800 rpm, 3 min), supernatant was removed followed by washing with Dulbecco’s phosphate-buffered saline (DPBS, Gibco Thermo Fisher Scientific) without (w/o) Ca^2+^ and Mg^2+^. Afterwards CBs were resuspended in dissociation medium (STEMcell Technologies). CBs were incubated for 5–10 min at 37 °C. Suspension was filtered through a 70 μm cell strainer and centrifuged again at 1000 rpm for 3 min. Pellet was resuspended in DPBS w/o Ca^2+^ and Mg^2+^ and dissociated cells were counted. Cell suspension for 3.9 or 5.0 × 10^6^ cells/mL was centrifuged at 1000 rpm for 3 min and cell pellet was resuspended in CryoStor^®^CS10 (STEMcell Technologies) or KSR (Gibco Thermo Fisher Scientific, mixed with 10µL/mL Pluronic-F68 (Gibco, Thermo Fisher Scientific), ROCK-inhibitor Y-27632 (10µM, Tocris Bioscience) and 10%DMSO (Sigma-Aldrich)) cryopreservation medium. Vials were placed into a controlled rate freezer (Planer Kryo 10 Series) for 1.5 h. Frozen cells were transferred to − 150 °C for storage. For thawing, cells were placed into a 37 °C water bath until only a small layer of ice was visible and then were directly and gently mixed with warm medium. Cells were handled and cultivated as described above. For CS10 medium we analyzed three vials and for KSR medium two vials.

### Determination of recovery rate

After thawing cells were counted by using 0.4% trypan blue exclusion (mixed 1:1, Sigma-Aldrich). Counted trypan blue negative cell number was subtracted from frozen cell number and calculated as recovery rate for each condition. Recovery rate = number of living cells post thaw/number of cells pre freeze.

### RNA-fluorescence in situ hybridization

Detection of active transcription sites (aTS) and cytoplasmic mRNA in hiPSC-CMs grown for 10 or 35 days on laminin-coated glass coverslips was performed as described previously^55^. Briefly, two probe sets for *MYH6*, *MYH7*,* MYBPC3* and *TNNI3*, respectively, were designed for detection of intronic and exonic sequences of each gene using the Stellaris^®^ Probe Designer (https://www.biosearchtech.com/support/tools/design-software/stellaris-probe-designer). Exonic sets were designed to hybridize with exonic sequences of respective mRNAs and were labelled with a Cy3-like fluorophore (Quasar 570, LGC Biosearch Technologies). Intronic sets were designed to hybridize with intronic sequences of respective pre-mRNAs and were labelled with a Cy5-like fluorophore (Quasar 670, LGC Biosearch Technologies). Following hybridization, active transcription sites were identified as co-localization of intronic and exonic signals inside nuclei of CMs. RNA-FISH procedures were performed according the manufacturer’s instructions (LGC BioSearch Technologies) with modifications. Medium was removed and cells were fixed for 20 min with 4% PFA (in DPBS, w/o Mg^2+^, Ca^2+^) at room temperature. Next, cells were washed three times with 1x DPBS (w/o Mg^2+^, Ca^2+^). Cells were then permeabilized for at least 1 h in 70% EtOH at 4 °C. Afterwards, cells were incubated in wash buffer (10% formamide, 2x saline-sodium citrate (SSC) in nuclease-free water) for 2–5 min, followed by over-night hybridization. Hybridization buffers (10% formamide, 10% dextran sulfate, 5% tRNA (20 mg/mL), 0.4% BSA (50 mg/ mL), 1% ribonucleosid vanadyl complex (200 mM), 10% 20xSSC in nuclease-free water)^56^ contained 125 nM of both, intronic and exonic probe sets per gene. Hybridization was performed in a sealed humidified chamber at 37 °C. After hybridization, cells were washed twice for 30 min with wash buffer at 37 °C with DAPI (80ng/mL, Sigma-Aldrich) in the second wash. Next, cells were incubated in 2xSSC for 2–5 min at RT and subsequently transferred from well plates into 10 µL GLOX anti-fade buffer (1 µL glucose oxidase (0.04 U), 1 µL Catalase (9.2 U), 10 µL Tris-HCL (1 M), 40 µL of 10% glucose, respectively, per ml 2xSSC) on microscope slides and stored on ice until imaging. Cells were imaged with an Olympus IX83 fluorescence microscope with a 60x oil objective (ApoN TIRFMN.A. 1.49, Olympus, Tokyo, Japan) and a metal halide light source. Images were recorded with a cooled CCD camera (Orca-R^2^, Hamamatsu, Photonics, Japan). Three-dimensional z-stacks were recorded with motorized shutter and z-stage using filter sets for DAPI (Chroma U-F4900, Chroma Technology Corp, Bellows Falls VT, USA), GFP (Chroma U-F49002), Cy3 (Chroma U-F49004) and Cy5 (Chroma U-F49006). Exposure times for DAPI were 20 ms, for GFP 250 ms, for Quasar 570 800 ms, and for Quasar 670 1 s. Images were taken from adjacent z-stacks separated by 0.3 μm. CellSens Dimension (version 2.3, Olympus, Tokyo, Japan) was used to count the number of active transcription sites. Cells were verified as CMs by fluorescence signals of sarcomeric gene mRNA in the cytoplasm. Cells without cytoplasmic mRNA signals were classified as non-CMs and were not analyzed. Transcriptional activity was determined by the number of aTS per nucleus.

### Immunofluorescence analysis of sarcomeric protein expression

For immunofluorescence (IF) staining of single hiPSC-CMs grown for 10 or 35 days on laminin-coated glass coverslips, cells were fixed with 4% PFA (Alfa-Aesar) in Dulbecco’s phosphate-buffered saline (DPBS) for 20–30 min at room temperature (RT). After fixation, hiPSC-CMs were washed three times in DPBS and permeabilized using 0.2% Triton-X-100 (Roche) for 15 min, followed by three additional DPBS washes. Blocking was performed in 5% bovine serum albumin (Gerbu Biotechnik GmbH) for 20 min to prevent nonspecific antibody binding. Primary antibodies against α-MyHC (rabbit, polyclonal, α-huMYH6, 1:50, BioGenes), β-MyHC (mouse, monoclonal, M8421, 1:100, Sigma-Aldrich, Merck) and cTnI (rabbit, polyclonal, 1:100, BioGenes) were incubated for 1 h at RT. As secondary antibodies, anti-rabbit Alexa Fluor 488 (goat, polyclonal, A11008, 1:400, Invitrogen Thermo Fisher Scientific) and anti-mouse Alexa Fluor 555 (goat, polyclonal, A21422, 1:400, Invitrogen Thermo Fisher Scientific) were used. Cells were incubated for 1 h at RT. Cells were washed each three times after incubation with antibodies in DPBS and DAPI (80ng/mL, Sigma-Aldrich) was used to stain nuclei. Only cells with sarcomeric striation pattern were analyzed by using 20x and 40x objectives, respectively. Using IF-staining, single hiPSC-CMs were categorized according to their level of α- vs. β-MyHC expression: exclusively β-MyHC expressing CMs, exclusively α-MyHC expressing CMs, and CMs with mixed α- and β-MyHC expression as shown previously^38^. For determination of percentage of cTnI-positive hiPSC-CMs, hiPSC-CMs as verified by β-MyHC or cTnT (mouse, monoclonal, MA5-12960, 1:100, Invitrogen Thermo Fisher Scientific) positive co-staining of sarcomeres were counted and the fraction of cells with a positive sarcomeric signal for cTnI was calculated. Immunofluorescence staining was used to determine the percentage of cardiomyocytes (positive sarcomere staining signal) vs. non-cardiomyocytes (no positive signal for sarcomere staining) per condition by counting cells in several randomly chosen fields of view.

### Morphology analysis of hiPSC-CMs

Immunofluorescence images of fixed hiPSC-CMs were used to measure cell length and width for calculation of aspect ratio as well as measuring sarcomere length. The aspect ratio was determined as the ratio of the maximum cell length to the maximum of cell width. For sarcomere length a minimum of 5 well aligned sarcomeres were measured and averaged. Fluorescence images were processed with a bandpass filter to blur sarcomeric striations and FibrilTool plug-in for ImageJ was used to assess the myofibrillar alignment^57^. For all analysis Image J/Fiji (National Institutes of Health, version 1.53c) was used.

### Analysis of twitch contraction parameters

Contractile properties of fresh and cryopreserved hiPSC-CMs grown for 8,9 or 10 (= day 10) and 35 or 36 days (= day 35) on laminin-coated glass coverslips were recorded by a video-based optical contraction analysis system (MyoCam, IonOptix, Milton, MA, USA) using the edge detection module. Shortly, coverslips with single adherent cardiomyocytes were placed in a custom-made perfusion chamber. CMs were electrically stimulated with the MyoPacer EP Cell Stimulator (IonOptix Corp.) via two platinum electrodes. Stimuli of supra-threshold voltage, 4 ms duration and a frequency of 1 Hz were applied. All experiments were performed at 37 ± 0.5 °C. Contractions were recorded by edge detection as described before^37,38^. For analysis 15–20 twitches per cell were averaged. Time to peak of shortening (ttp), time from peak to 50% relaxation (half relaxation time, hrt), contraction amplitude, maximum contraction (shortening) velocity and maximum relaxation velocity were determined using IonWizard software (IonOptix Corp., Milton, MA, USA, version 6.5). Spontaneous beating was observed in all conditions but not recorded, instead we focused on analysis of hiPSC-CMs responding to 1 Hz pacing. Only a few CMs in all conditions were arrhythmic and not recorded.

### Statistics

Statistical analyses were performed with GraphPad Prism 9.5.1. Data were first tested for normality using Shapiro-Wilk test. Differences of contraction parameters between fresh and cryopreserved hiPSC-CMs were analyzed by Mann-Whitney U test. Unpaired t test was used for recovery rate. One-way ANOVA was used for percentage of CMs and fraction of exclusively β-MyHC and cTnI, respectively, expressing hiPSC-CMs as well as for comparison of aTS per nucleus. Kruskal-Wallis test was performed for morphology analysis. Significant differences were indicated with *p* < 0.05. Two to four cover slips and up to 16 for contraction analysis were analyzed per analysis time point and condition.

## Data Availability

The data that support the findings of this study can be directed to the corresponding author Kathrin Kowalski ( [Kowalski.kathrin@mh-hannover.de](mailto: Kowalski.kathrin@mh-hannover.de) ) upon reasonable request.
